# Aquatic–terrestrial linkages drive contrasting biodiversity patterns in tropical and temperate forests

**DOI:** 10.1098/rspb.2024.2423

**Published:** 2025-01-08

**Authors:** Liam N. Nash, Fátima C. Recalde, Timothy Chambers, Victor S. Saito, Gustavo Q. Romero, Pavel Kratina

**Affiliations:** ^1^ Centre for Biodiversity and Sustainability, School of Biological and Behavioural Sciences, Queen Mary University of London, Mile End Road, London E1 4NS, UK; ^2^ Laboratory of Multitrophic Interactions and Biodiversity, Department of Animal Biology, Institute of Biology, University of Campinas (UNICAMP), Campinas, São Paulo 13083-862, Brazil; ^3^ Environmental Sciences Department, Federal University of São Carlos, São Carlos, São Paulo 13565-905, Brazil

**Keywords:** aquatic–terrestrial linkages, beta-diversity, community analysis, riparian biodiversity, spiders, tropical–temperate comparisons

## Abstract

Riparian ecosystems harbour unique biodiversity because of their close interconnections with adjacent aquatic ecosystems. Yet, how aquatic ecosystems influence terrestrial biodiversity over different spatial scales is poorly understood, particularly in the tropics. We conducted field campaigns to collect 235 terrestrial invertebrate assemblages along 150 m transects from 47 streams in both Brazil and the UK, compiling one of the largest known datasets of riparian invertebrate community composition at multiple spatial scales. Invertebrate densities increased towards water in both regions. In Brazil, this was driven by an increase in spiders, with a corresponding decrease in non-predators, resulting in higher predator : prey ratios near water. In the UK, non-predator densities increased towards water, decreasing predator : prey ratios. While pairwise dissimilarity increased with distance from water in both regions, β-diversity was significantly higher in tropical assemblages, with more β-diversity explained by turnover. Spider community composition was significantly structured by distance from water in the Brazilian sites, suggesting tropical assemblages were influenced more by emerging aquatic prey, with a distinct spider community replacing other predators, with possible top–down control of terrestrial prey. High turnover-driven dissimilarity among tropical assemblages suggests that Brazilian riparian ecosystems are better managed at the landscape scale, with an emphasis on in-stream measures preventing disruption of aquatic resource subsidies.

## 1. Introduction

Aquatic ecosystems have powerful impacts on surrounding riparian ecosystems, influencing terrestrial environments beyond their hydro-geomorphic boundaries [1,2]. The specific biotic and abiotic conditions arising from proximity to water, such as disturbance from flooding and drought, microhabitat and vegetation gradients, elevated water and nutrient availability and cross-ecosystem resource flows, contribute to riparian habitats having unique communities relative to upland habitats [3] and increasing regional diversity [4]. These influences are expected to vary with distance from water, across catchments and among regions, depending on local characteristics of both aquatic and terrestrial ecosystems [3]. However, we still lack a corresponding understanding of how terrestrial riparian biodiversity is organized over space in response to spatially distributed aquatic influences [5].

A detailed understanding of aquatic–terrestrial connections is key for landscape-level riparian management [6], such as evaluating pollutant pathways [7] or determining riparian buffer zone width and management strategies [8]. While riparian buffers are generally implemented to protect waterways from terrestrial anthropogenic influences, such as agricultural run-off and bank-erosion, they are increasingly regarded as an important habitat for terrestrial biodiversity [9]. Thus, many buffers, typically 5–50 m, are not considered wide enough for effective terrestrial biodiversity conservation [9]. In the UK, 1 m buffers for waterway protection are mandatory, while buffers of 10–50 m for other ecosystem services, including terrestrial biodiversity provision, are only recommended [10]. Furthermore, the spatial extent of aquatic influences in the terrestrial environment is generally overlooked [8].

Under the ‘meta-ecosystem’ concept, cross-ecosystem fluxes of resources and organisms provide the link that scales from local community processes to landscape dynamics [11]. One of the most significant aquatic–terrestrial fluxes is through aquatic insect emergence [12]. The spatial extent to which this resource flux impacts terrestrial biodiversity is determined by the magnitude of emergence, barriers to aquatic prey dispersal [1] and the characteristics of terrestrial consumers [12]. As aquatic insects are often of higher nutritional quality than terrestrial prey [13], and their supply is donor-controlled (i.e. unaffected by consumption), they subsidize predator populations, modifying top–down forces on other riparian biodiversity. Generalist predators bolstered by aquatic resources may suppress local prey populations [14], while specialist predators may release local prey from predation pressure through prey-switching [15]. Describing the spatial distribution and composition of riparian predators not only improves understanding of where to prioritize riparian management but also how aquatic influences are distributed throughout the terrestrial community. Despite this, most previous research has focused on terrestrial communities closely adjacent to water at a single spatial scale, often overlooking catchment, landscape or global heterogeneity [1,2].

There is a particular lack of studies on riparian biodiversity and aquatic–terrestrial linkages from the tropics [1,3,16]. This is despite key differences between tropical and temperate ecosystems, such as higher overall terrestrial biodiversity [17], greater variation in the magnitude of flooding arising from heavy, variable precipitation [18] and higher stochasticity in community assembly due to faster metabolic rates and shorter generation times [19]. Tropical ecosystems are expected to respond differently to aquatic influences than temperate ecosystems because of high primary productivity [20], higher predation rates [21], less seasonal aquatic insect emergence [22] and higher aquatic resource use [16]. Although the condition of tropical buffer habitat can vary, with resulting impacts on species recovery [23], several tropical countries have larger riparian buffer requirements than seen in the UK (e.g. Brazil: 5–100 m) and terrestrial biodiversity is more likely to be considered in buffer policies [8]. Yet, a deficit of research from tropical regions means tropical riparian protection policies are often based on research conducted in temperate regions [8]. Tropical freshwater ecosystems, including their interlinked terrestrial riparian surroundings, are under major pressure from rapidly changing human land-use [24,25]. A rigorous comparative approach is needed to alleviate these biases in global research distribution by facilitating direct comparisons between tropical and temperate studies.

We aimed to determine the spatial extent to which stream ecosystems influence terrestrial communities in a tropical region (Brazil) and a temperate region (UK). We conducted field sampling to compare patterns of community composition, abundance, species richness (α-diversity), and using β-diversity, community dissimilarity at different spatial scales. Aquatic ecosystems may increase terrestrial β-diversity by supporting higher numbers of individuals or species or by supporting unique species compositions (turnover), with important management implications [26]. We hypothesized that community dissimilarity would increase with distance due to environmental filtering and dispersal limitation [27,28]. However, distance from water should constrain this variation so that assemblages are more similar to each other at similar distances from water, even if from different streams, because of influences from the aquatic environment [4].

Previous research of invertebrate predators at our Brazilian sites showed they incorporate higher proportions of aquatic prey in their diets than the predators at our UK sites [16]. Because of this, we expected the tropical assemblages to have stronger lateral gradients of biodiversity patterns with distance from water, especially for spiders, known interceptors of aquatic prey. Alternatively, greater preference for aquatic prey by tropical predators could dampen biodiversity gradients, as predators select aquatic prey even as it becomes scarce away from water. Furthermore, if alternative gradients of aquatic influence, such as vegetation structure and density, microclimate or topography, are the dominant drivers of riparian biodiversity over aquatic prey inputs, we expected lateral biodiversity gradients would be similar among spiders, other predators and non-predators, and not necessarily correlate with aquatic resource use. We hypothesized higher regional riparian richness, but lower abundances, in the Brazilian sites than in the British sites [29], because faster metabolic rates, shorter development times and more efficient energy transfer reduce standing densities in the tropics [19,30]. We also predicted higher β-diversity at the Brazilian sites because low abundances combined with high regional diversity are predicted to increase stochastic differences between local communities [19,30].

## 2. Methods

### (a) Sampling design and collection

We conducted field campaigns to sample terrestrial invertebrate communities within forested riparian zones, at multiple, hierarchically nested, spatial scales, following the protocol from a previous study [16]. We systematically sampled four tropical sites in Brazilian rainforests and three temperate sites in British mixed deciduous coniferous woodland (electronic supplementary material, appendix S1, table S3), representing two regions with divergent climates and riparian buffer policies. Sites in both regions were within protected areas to reduce anthropogenic variation among replicates. At each site, we aimed to sample seven streams (between 0.5 and 3 m wide), although at two sites we could only sample six streams, due to stream availability and access within the same catchment. Sampling was conducted between 2016 and 2021, with each individual site sampled within a limited timeframe (mean 8.8 days) to control for temporal variation among streams within the same site. To further reduce seasonal variation, sampling was conducted during the season of highest aquatic insect emergence in both regions: the rainy season for Brazilian sites (September–March), and late spring–early summer for British sites (May–July).

We sampled terrestrial invertebrates associated with vegetation at five distances (1, 10, 30, 60 and 150 m) from water along 150 m transects (based on average aquatic insects’ dispersal limits [31]) running perpendicular from the streambank into an area of continuous forest. All sampling was at least 150 m away from the sampling locations of neighbouring studies (see [16]), other water sources and the forest border in all directions, to eliminate pseudoreplication and edge effects. In total, we sampled terrestrial invertebrate assemblages along transects at 235 points from 47 streams across the seven sites and two regions. At each designated distance from water, we collected vegetation from ground level to 2 m high, within a 2 m radius. Following [14], we carefully enclosed the foliage within 50–70 l plastic bags and cut the stems at the bag edge before sealing, ensuring all material was well within the bags. Care was taken to minimize dense, woody sections of vegetation to avoid biasing the vegetation mass. Vegetation was sampled until 0.6 kg mean wet plant biomass was collected from a variety of plant species (weighed using a handheld Pesola 1 kg light-line spring-scale) to provide a representative sample of the terrestrial invertebrates associated with vegetation. Each bag was weighed to correct for sampling effort and to express invertebrate abundance and richness as densities per kilogram of plant biomass. Under laboratory conditions, all invertebrates visible without visual aids were collected from the surface of the vegetation and fixed in 70% ethanol. Using stereo-microscopes, we identified individuals based on morphology to family level or the lowest taxonomic unit necessary to separate predators from non-predators, before sorting into morphospecies at each site (see electronic supplementary material, table S1 for a full species and morphospecies list per site and table S2 for a summarized overview per region). We contrasted riparian invertebrate predator versus non-predator biodiversity patterns to determine the effects of aquatic prey subsidies on predators and the wider riparian community. The proportion of aquatic prey in the predator diets at our study sites was determined in a previous study using stable isotope analysis [16]. This study found that predators at the Brazilian sites consumed significantly more aquatic insect prey than at the British sites (27% more, on average), and this declined with distance from water. Any terrestrial adult aquatic insects collected for this present study (2% of individuals) were considered part of the terrestrial community and not as a measure of aquatic prey use by terrestrial predators. We compared the response of spider-only assemblages with the overall predator community (including spiders with 13 other predatory taxa; see electronic supplementary material, table S2 for a list of orders) because spiders are major interceptors of aquatic prey [16] and likely respond more strongly to aquatic resource inputs than other predators [20,32,33].

### (b) Density, richness and β-diversity metrics

To determine the influence of aquatic ecosystems on terrestrial assemblages across multiple spatial scales, we analysed abundance (as individuals per kilogram of vegetation sampled), richness (α-diversity, morphospecies per kilogram of vegetation sampled) and dissimilarity (β-diversity). To compare assemblage dissimilarity at different spatial levels, we calculated multi-sample total β-diversity (*BD*_total_), based on the total variation in a community matrix [34]. *BD*_total_ was calculated from abundance-based Bray–Curtis dissimilarity indices, to give less weight to rare species and incorporate our effort adjustment (abundance per kilogram of vegetation). To determine the dominant components of β-diversity at different spatial scales, we used a Bray–Curtis-based index from the Podani family (or ‘percentage difference’ [35]) to decompose *BD*_total_ into taxonomic turnover (*Rep*l_%diff_: dissimilarity driven by species replacement) and abundance differences (*AbDif*_%diff_: dissimilarity driven by numerical changes). As *Repl*_%diff_ *+ AbDif*_%diff_ = *BD*_total_*,* each component was expressed as a proportion of *BD*_total_, to determine their relative contribution to overall β-diversity [35].

### (c) Change in diversity with distance from water

To determine whether β-diversity changes with a distance from water, we compared individual pairwise Bray–Curtis dissimilarities between each assemblage along an individual transect with the reference stream-side assemblage at 1 m. To determine whether β-diversity change over distance from water was driven by turnover or abundance changes, each pairwise dissimilarity value was decomposed into *Repl*_%diff_ and *AbDif*_%diff_ and expressed as a proportion of *BD*_total_.

To determine how terrestrial community structure and diversity are affected by increasing distance from water between regions, we applied three linear mixed-effects models (LMEs) with abundance, α-diversity, predator-to-non-predator ratios (both abundance and richness) and β-diversity (the pairwise dissimilarity from this given assemblage relative to the stream-side assemblage) as response variables, for the entire invertebrate predator community (including spiders and other predatory taxa), spiders-only and non-predatory invertebrates. Densities were ln-transformed to meet assumptions of homoscedasticity and normality of model residuals, as verified with QQ plots and residual plot diagnostics. All LMEs included the interactive and main effects of distance from the stream (1–150 m; or distance from the reference assemblage for β-diversity: 10–150 m) and region (tropical, temperate). Stream ID nested within the sampling site was included as a random effect to account for variation in baseline diversity between sampling locations and years. LMEs are more robust to unbalanced designs (in this case, three British versus four Brazilian sites) than other model types. We compared random effect structure combinations using Akaike’s information criterion [36], corrected for small sample sizes (AICc), keeping the fixed effects constant with restricted maximum likelihood parameter estimation [37]. We selected the best random effect structure as that with the smallest AICc score, distinguishing among models when ΔAICc > 2, for model simplification of the fixed effects [37]. We then tested the main effects with maximum likelihood parameter estimation using deletion tests, based on Satterthwaite’s approximation for degrees of freedom, over likelihood ratio tests, as it reduces type I error likelihood for uneven data structures [38]. If the distance-region interaction was non-significant it was removed from the model before retesting the main effects independently. Model goodness of fit, set back to using restricted maximum likelihood parameter estimation, was gauged by the R^2^_conditional_, explaining variation from both mixed and random effects, and R^2^_marginal_, explaining variation based on the main effects alone [39].

### (d) Community composition

We also evaluated community compositional changes with distance from water using partial distance-based redundancy analysis (db-RDA), on square-rooted Bray–Curtis dissimilarities, assuming a linear response to distance from water [40,41]. We used invertebrate family abundances to analyse temperate and tropical sites in single models, as morphospecies classification systems varied among sites. For each invertebrate group (all-predators, spiders-only, non-predators) and region (tropical, temperate), we constructed db-RDA models with distance from the stream as a continuous effect, and stream identity nested within the site as partial conditional factors, to control for baseline variation between sites [42]. The significance of the overall model, the individual db-RDA axes and the effect of distance from water on community composition were determined using permutation tests with 999 permutations.

### (e) Dissimilarity at multiple spatial scales

For each site, we calculated *BD*_total_ at four spatial scales (electronic supplementary material, appendix S1, figure S1c): (i) β_1_, ‘within-site’ overall dissimilarity among all assemblages from each site; (ii) β_2_, ‘between-stream’ dissimilarity among assemblages along a single transect from the same stream; (iii) β_3_, ‘within-stream’ dissimilarity among assemblages at different distances from the same stream (calculated per stream and averaged); and (iv) β_4_, ‘within-distance’ dissimilarity among assemblages at the same distance from water but at different streams (calculated per distance and averaged). We applied ANOVA to test whether (i) *BD*_total_ and (ii) the proportion of *BD*_total_ governed by turnover (*Repl*_%diff_), varied among spatial scales (β_1_, β_2_, β_3_, β_4_), and whether this differed between regions and among groups. We used post hoc Tukey testing to determine significant differences between factor-pairs of significant effects.

All statistical analyses were carried out in the R statistical software (v. 4.2.3, R Foundation for Statistical Computing, 2023), using the ‘capscale’ and ‘anova.ccc’ functions from the *vegan* package [43] for db-RDA fitting and evaluation; the ‘beta.div.comp’ function to calculate BD_total_, *Repl*_%diff_ and *AbDif*_%diff_ from the *adespatial* package [44]; *lme4* [45] and *lmerTest* [38] to construct and test LMEs and *MuMIn* [39] for R^2^_cond._ and R^2^_marg._ calculations.

## 3. Results

### (a) Overall density and richness

**Figure 1.**
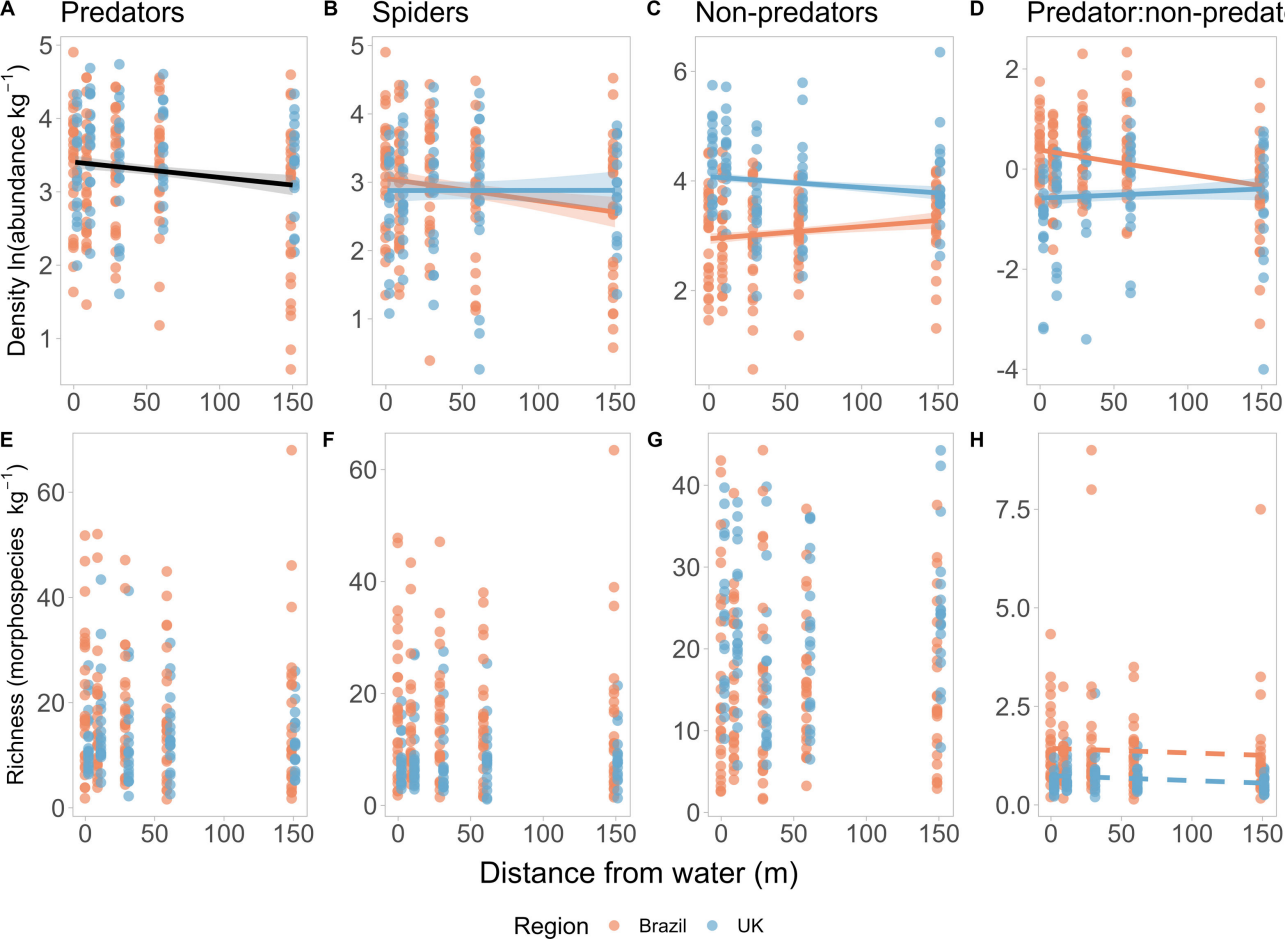
The individual density (A-D) and morphospecies richness (E-H) of invertebrate (A,E) predators (including spiders and 13 other predatory taxa), (B,F) spiders only, (C,G) non-predators**,** as well as the ratio between predator and non-predator density (D) and richness (H), with increasing distances from water (1–150 m), in British (blue) and Brazilian (orange) sites. Density is the ln-abundance per kilogram vegetation sampled, while richness is the number of morphospecies per kilogram vegetation sampled. Regression lines and 95% confidence intervals are drawn using the *predict*() R function, based on final LME models, coloured black when no significant difference between regions was detected but distance was still significant, or coloured by region when a significant intercept (dashed line) or interactive difference (solid line) was detected. All models included stream ID nested within site as a random effect to control for variation between sampling locations.

Across all 235 terrestrial invertebrate assemblages sampled, we collected 11 861 individuals from 140 kg of riparian vegetation, from 27 major invertebrate groups, including 16 insect and 4 arachnid orders (electronic supplementary material, appendix S1, figure S2a, table S1). Spiders were the most abundant predators in both the tropical Brazilian (78.4%) and temperate UK (59.6%) sites ( electronic supplementary material, appendix S1, figure S2b, table S2). Total invertebrate density was higher in the temperate (mean = 118.87 invertebrates per kilogram) than in the tropical assemblages (mean = 62.55 invertebrates per kilogram). This was driven by significantly greater densities of invertebrate non-predators (table 1; figure 1*c*) compared with predators, which were similarly dense between regions (figure 1*a*), resulting in significantly higher predator : prey densities in the Brazilian assemblages (mean = 1.63) compared with the British assemblages (mean = 0.87; figure 1*d*). Assemblage morphospecies richness per kilogram (α-diversity) was similar between regions for both predators (table 1; figure 1*e*) and non-predators (figure 1*g*), but Brazilian α-diversity was predator-dominated with significantly higher predator : prey richness per kilogram than the UK assemblages (figure 1*h*).

**Table 1.** Summary statistics of linear mixed effects model selection, evaluation and analysis. Multiple metrics of terrestrial community structure and diversity: density (ln-abundance kg^−1^), richness (morphospecies kg^−1^), β-diversity (pairwise Bray–Curtis dissimilarity relative to stream-side assemblage) and turnover (as a proportion of total β-diversity, relative to stream-side assemblage) were analysed separately for the entire predator, spider-only and non-predator invertebrate assemblages, as well as the ratio of predators to non-predators (for density and richness only). Maximum models were simplified by first comparing between random effect structures using Akaike’s information criterion (AICc): ‘1|' indicates a random intercept model and ‘site/stream’ indicates nested random effects. Once the best random effects structure was selected with restricted maximum likelihood parameter estimation, model simplification of the fixed effects with maximum likelihood parameter estimation was carried out, including for regions (tropical and temperate), distance from stream for density and richness (1–150 m) or from the reference assemblage for β-diversity metrics (10–150 m) and the interaction between them (region × distance). Values in bold represent parameters retained in the final model when *p *< 0.05. F-statistics and *p*-values were calculated using Satterthwaite’s approximation for degrees of freedom. The goodness of fit of the reduced model was evaluated using the marginal and conditional R^2^.

*metric*		explanatory variables						random effect structure		goodness of fit
group		region		distance		region × distance		1| site/stream	1| site	
		f (d.f.)	*p*	f (d.f.)	p	f (d.f.)	*p*	AICc (d.f.)	AICc (d.f.)	R^2^_m_, R^2^_c_
***density***										
**predators**		0.64	0.448	**8.85**	**0.003**	2.72	0.101	**498.67**	507.63	0.05, 0.53
		(1, 7)		(**1, 188**)		(1, 188)		(**7**)	(6)	
**spiders**		0.003	0.962	**7.25**	**0.008**	**3.95**	**0.048**	**520.89**	526.23	0.02, 0.61
		(1, 7)		(**1, 186.1**)		(**1, 186.5**)		(**7**)	(6)	
**non-predators**		**12.34**	**0.01**	0.24	0.624	**6.13**	**0.014**	**571.22**	576.37	0.24, 0.48
		(**1, 7.1**)		(1, 187.7)		(**1, 187.5**)		(**7**)	(6)	
**predator : non-predator**		**5.72**	**0.047**	**4.7**	**0.031**	**9.13**	**0.003**	**633.39**	635.66	0.12, 0.34
		(**1, 7.2**)		(**1, 187.9**)		(**1, 187.7**)		(**7**)	(6)	
***richness***										
**predators**		0.38	0.557	2.42	0.122	0.16	0.687	**1651.76**	1660.58	0.03, 0.64
		(1, 7)		(1, 185.7)		(1, 185.6)		(**7**)	(6)	
**spiders**		1.65	0.24	2.72	0.101	1.07	0.303	**1542.12**	1565.06	0.09, 0.73
		(1, 7)		(1, 175)		(1, 175)		(**7**)	(6)	
**non-predators**		2.04	0.196	0.27	0.604	1.59	0.208	**1673.61**	1680.03	0.08, 0.51
		(1, 7)		(1, 187.6)		(1, 187.4)		(**7**)	(6)	
**predator : non-predator**		**11.38**	**0.012**	1.08	0.3	0.02	0.878	695.3	**693.17**	0.1, 0.17
		(**1, 7**)		(1, 226.1)		(1, 226.1)		(7)	**(6)**	
***β-diversity***										
**predators**		**6.94**	**0.033**	**6.59**	**0.011**	1.59	0.21	**−182.01**	−168.59	0.23, 0.64
		(**1, 7**)		(**1, 140**)		(1, 139.8)		(**7**)	(6)	
**spiders**		**7.25**	**0.031**	**5.13**	**0.025**	1.87	0.173	**−146.79**	−140.57	0.24, 0.61
		(**1, 7**)		(**1, 136.7**)		(1, 136.6)		(**7**)	(6)	
**non-predators**		**9.99**	**0.017**	**6.71**	**0.011**	3.45	0.065	**−170.93**	−168.12	0.14, 0.34
		(**1, 6.6**)		(**1, 141**)		(1, 140.8)		(**7**)	(6)	
***turnover proportion***										
**predators**		0.07	0.801	0.01	0.937	**8.08**	**0.005**	**30.84**	36.72	0.03, 0.29
		(1, 7)		(1, 138.4)		(**1, 138.3**)		(**7**)	(6)	
**spiders**		**3.75**	**0.055**	1.35	0.247	0.06	0.806	65.34	**64.87**	0.03, 0.03
		(**1, 180**)		(1, 180)		(1, 180)		(7)	(**6**)	
**non-predators**		1.05	0.311	0.08	0.781	0.1	0.755	**45.44**	62.54	0.01, 0.33
		(1, 46.8)		(1, 140.4)		(1, 140.3)		(**7**)	(6)	

### (b) Changes in density and diversity with distance from water

Total predator density significantly declined with distance from water in both the Brazil and UK sites (table 1; figure 1*a*). However, when spiders were analysed alone, the tropical spider density declined away from water, whereas the temperate spider densities remained similar with distance from water (table 1; figure 1*b*). Moreover, the tropical non-predator densities increased, while the temperate non-predator densities decreased with distance from water (table 1; figure 1*c*). Due to the numerical dominance of spiders in the predator assemblage, tropical predator-to-non-predator density ratios were higher near water, compared to temperate ratios that increased away from water (table 1; figure 1*d*). Morphospecies richness per kilogram (α-diversity) was similar with distance from water in the all-predator (figure 1*e*), spider-only (figure 1*f*) and non-predator (figure 1*g*) assemblages (table 1).

**Figure 2.**
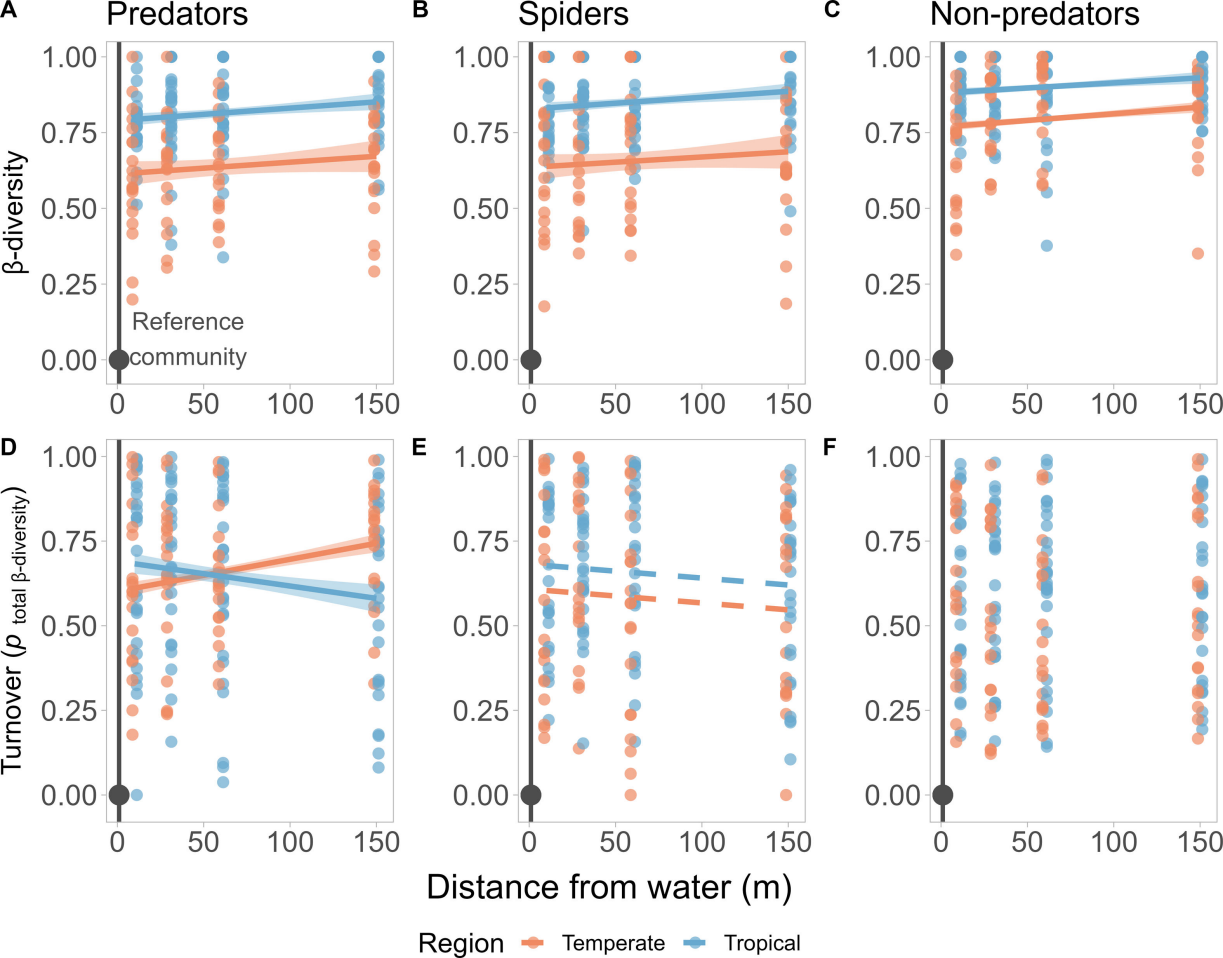
Changes in β-diversity with increasing distance from water expressed as the pairwise Bray–Curtis dissimilarity between the stream-side reference assemblage at 1 m (dark grey point and line) and assemblages further along the transect (A-C), and the proportion of β-diversity driven by species turnover (D-F), for all predators (A,D), spiders-only (B,E) and non-predators (C,F). Regression lines and 95% confidence intervals are drawn using the *predict*() R function, based on final LME models, coloured by region when a significant intercept (dashed line) or interactive difference (solid line) was detected. All models included the site as a random effect to control for variation between sampling locations.

The pairwise dissimilarity (β-diversity) between the reference assemblage 1 m from water and those further along each transect significantly increased with distance for the all-predator (figure 2*a*), spider-only (figure 2*b*) and non-predator (figure 2*c*) assemblages (table 1). β-Diversity between the stream-side assemblage and those further away from water were consistently higher in tropical sites, for all functional groups (table 1). For predators overall, this was driven by opposing components of β-diversity depending on the region (table 1; figure 2*d*), with turnover increasing with distance in the British sites, but declining, relative to abundance differences, in Brazil. The contribution to β-diversity from turnover relative to abundance differences was greater in the tropical spider assemblages compared with the temperate spiders (figure 2*e*), but this did not change with distance from water (table 1). There was no significant difference in contribution of turnover to β-diversity between Brazilian or British non-predator assemblages (figure 2*f*), and this did not vary with distance from water (table 1).

### (c) Community composition

**Figure 3.**
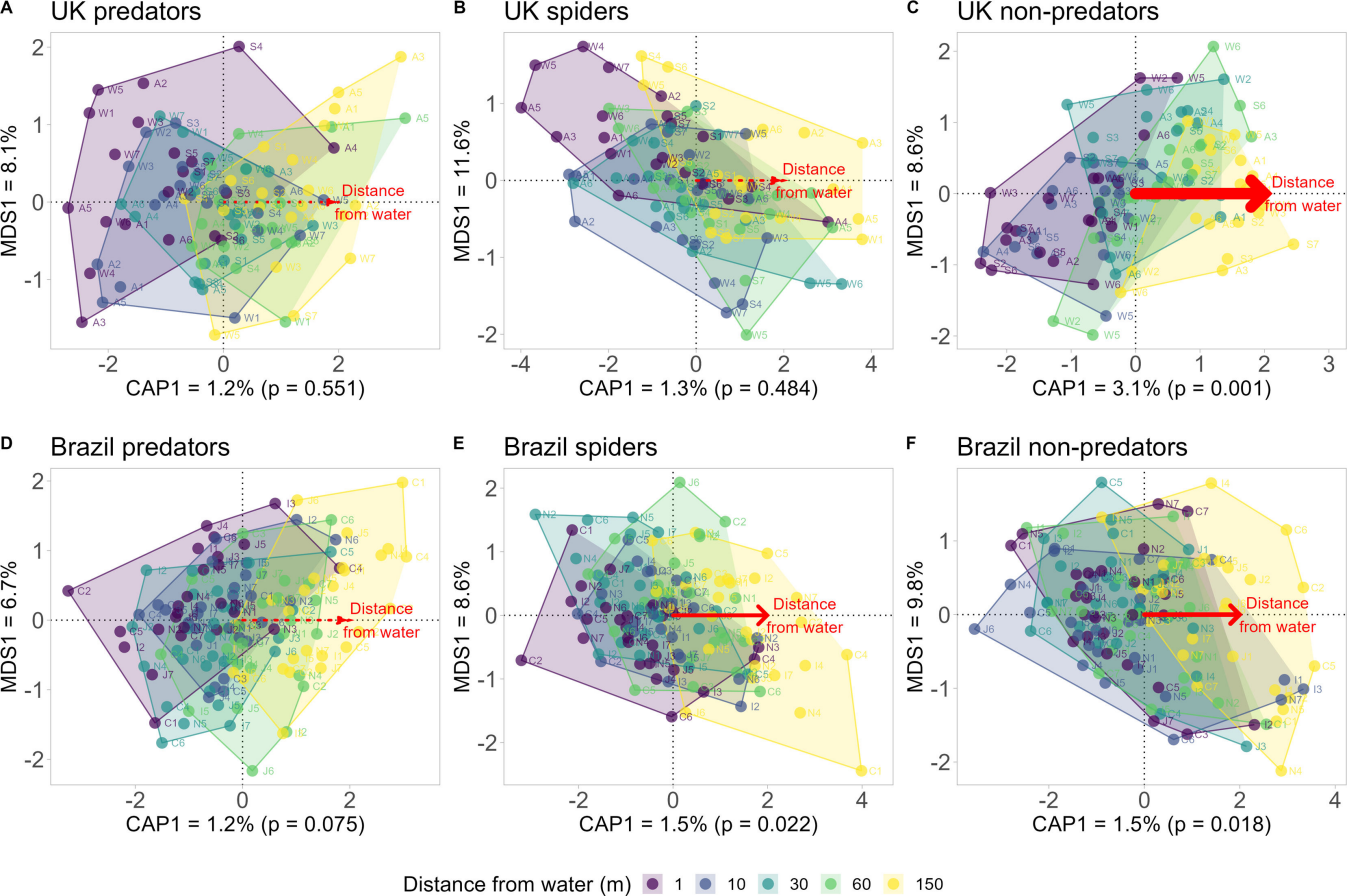
Distance-based redundancy analysis showing compositional shifts with distance from water of (A,D) all predators, (B,E) spiders only and (C,F) non-predators between (A-C) tropical Brazilian and (D-F) temperate British sites. Each point represents an assemblage of invertebrate families. Solid red arrows represent significant relationships based on ANOVA-like permutation tests with 999 permutations. Arrow width is proportional to the variation explained (R^2^) by distance from water. All db-RDA models controlled for stream ID nested within the site using conditional factoring, illustrated using point labels with the first character representing the site (A = Ashdown, S = Scotland, W = Wales, C = Cananéia, J = Serra do Japi, I = Iguacu, *n* = North Amazon) and the second representing stream ID. CAP1 and MDS1 represent the first constrained and unconstrained axes used in the analysis.

For all predators, the family composition of their assemblages was not significantly structured by distance from water in either Brazil (db-RDA: F_1, 105_ = 1.248, *p* = 0.106; figure 3*a*) or the UK (db-RDA: F_1, 79_ = 0.948, *p* = 0.577; figure 3*d*). However, when spiders were considered alone, the tropical spider assemblages were significantly structured along the 150 m gradient from water (db-RDA: F_1, 101_ = 1.571, *p* = 0.018; figure 3*c*), while the temperate spider assemblages were not (db-RDA: F_1, 77_ = 0.979, *p* = 0.473; figure 3*f*). By contrast, non-predator assemblage composition was significantly structured along the 150 m gradient from water in both Brazilian (db-RDA: F_1,106_ = 1.607, *p* = 0.014; figure 3*b*) and British sites (db-RDA: F_1, 79_ = 2.495, *p* = 0.001; figure 3*e*).

### (d) β-Diversity at multiple spatial scales

**Figure 4.**
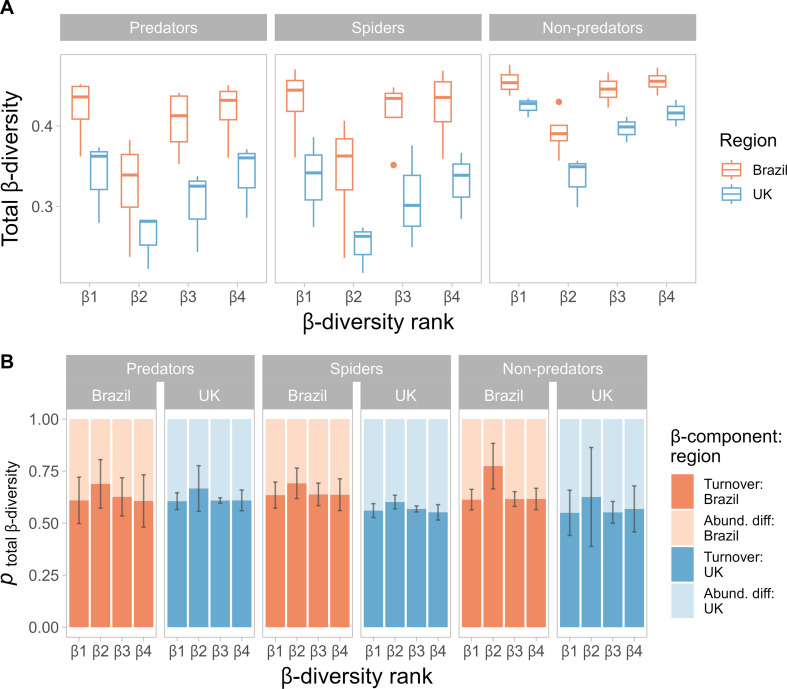
Temperate British (blue) and tropical Brazilian (orange) patterns of terrestrial riparian diversity of all predators, spiders-only and non-predators analysed alone (calculated per site) across different scales. (A) Multi-sample total β-diversity (BD_total_) was calculated from the overall variance in a Bray–Curtis dissimilarity matrix at four scales: β_1_ ‘within-site’, β_2_ ‘between-stream’, β_3_ ‘within-stream’ and β_4_ ‘within-distance’ dissimilarity. (B) At each of these scales, the total β-diversity was divided into the proportion governed by species turnover (Repl_%diff_) versus abundance differences (AbDif_%diff_). All metrics were calculated per site and analysed per region. Error bars represent the regional mean ± s.d.

The total β-diversity (BD_total_) was significantly higher in Brazil compared with the UK (ANOVA: F_1, 66_ = 68.15, *p* < 0.001; figure 4*a*), independently of functional group (ANOVA: F_2, 66_ = 3.1, *p* = 0.051) and spatial scale (ANOVA: F_3, 66_ = 0.2, *p* = 0.895). Assemblages among streams (β_2_) were significantly less dissimilar to each other than the overall site-level dissimilarity among all local assemblages (β_1_; Tukey: *p* < 0.001). Overall dissimilarity among all local assemblages across each site (β_1_) was not significantly different to dissimilarity between assemblages at different distances from the same stream (β_3_; Tukey: *p* = 0.377) or assemblages at the same distance from water but at different streams (β_4_; Tukey: *p* = 0.994). Turnover was the most important component of total β-diversity across all sites and groups, relative to abundance differences (figure 4*b*). However, the proportion driven by turnover was significantly higher in Brazil than in the UK (ANOVA: F_1, 66_ = 9.32, *p* = 0.003), but did not differ among all invertebrate predators (including spiders), non-predators and spiders-alone (ANOVA: F_1, 66_ = 0.155, *p* = 0.856). Relative turnover was significantly higher between streams (β_2_) than across all assemblages (β_1_; (Tukey: *p* = 0.013), among assemblages at different distances at the same stream (β_3_; Tukey: *p* = 0.026) and among assemblages at the same distance from water but at different streams (β_4_; Tukey: *p* = 0.016).

## 4. Discussion

Through our field campaigns, collecting one of the largest known datasets of riparian invertebrate assemblages, we demonstrate that riparian biodiversity is highly spatially structured with distance from aquatic ecosystems following declining aquatic influences (see [16]). Despite high heterogeneity within sites, we found distinct biodiversity patterns between the Brazilian and British sites. Nevertheless, in both regions, we show that riparian communities sustained higher densities of predators in proximity to water, and were often compositionally distinct, but not more species rich. These results support the concept that riparian ecosystems increase regional diversity by housing unique, rather than more, species [4]. How riparian predator versus non-predator assemblages were structured with respect to the aquatic environment, and how aquatic ecosystems may indirectly influence other components of terrestrial food webs, strongly depended on the region. The divergent responses between communities in the UK and Brazil have distinct implications for the management and protection of riparian biodiversity.

Aquatic ecosystems had a positive and homogenizing influence on predator assemblages, which became denser and more similar with increasing proximity to water in both regions. However, in Brazil, relative turnover among predator assemblages closer to water became higher. β-diversity is predicted to decrease with abundance in regions with weak selective forces, as the probability of matching species between assemblages increases [30]. Increasing turnover near water, even with increasing abundance, suggests that the aquatic ecosystems in tropical Brazil are supporting a larger species pool near water, reducing the probability of matching species in larger communities [30]. Although the temperate predator assemblages were more similar to each other near water, following expected distance–dissimilarity relationships [46], this was predominantly driven by changing abundance, suggesting aquatic ecosystems in the temperate UK support greater densities of individuals but not necessarily a larger species pool.

Increasing predator turnover at the Brazilian sites was likely driven by the replacement of non-spider predators with a distinct spider assemblage near water. While the overall tropical predator community was not distinctly structured by distance from water, spider assemblages changed consistently along this gradient. In the UK sites, neither the whole nor spider-only predator communities changed with distance from water. The temperate spiders had similar densities with distance, with the increase in predator abundance evidently driven by other predatory taxa [3]. A sister study using stable isotope analysis on riparian invertebrates (following an identical sampling design of invertebrates collected along different transects) demonstrated significantly higher aquatic insect resource use in spiders from the Brazilian sites than the British sites [16]. This suggests that aquatic-derived insect subsidies are a dominant driver of the stronger community response of the tropical spiders to aquatic proximity, relative to other predators, observed here. Several alternative riparian gradients may have contributed to the observed diversity patterns, influencing spiders more than other predators, such as vegetation structure and composition, microclimate or underlying soils [3]. For example, tropical spiders were shown to respond positively to riparian vegetation providing substrate for web attachment [47]. Further research directly measuring different gradients of aquatic influence within the same analysis would facilitate the determination of the relative importance of different mechanistic drivers of riparian predator patterns.

Whereas non-predator density in Brazil declined towards water, in the UK it increased. In combination with the contrasting relationships of tropical and temperate spiders, this resulted in strongly divergent predator-to-non-predator density ratios, with predators numerically dominating riparian assemblages towards water in the tropical sites. Spiders are typically generalists [20], and tropical spiders from these communities have diverse diets, utilizing terrestrial prey alongside high quantities of aquatic prey [16]. Generalist predators subsidized by aquatic resources can strongly control terrestrial prey due to apparent competition with aquatic prey [48,49]. Our results suggest how, in some tropical areas, high but declining aquatic resource use with distance from water, may lead to a spatial gradient of top–down impacts on terrestrial communities through correlated numerical and community responses of spiders. In Brazil, changes in non-predator community structure may have been partly influenced by a predator-mediated indirect effect of aquatic resource subsidies. However, in the UK despite an increase in non-predator density towards water, predator-to-non-predator density ratios remained similar, suggesting that temperate non-predator communities are structured by other gradients, such as riparian vegetation diversity or water availability [3]. As with the predator results, future research incorporating such additional riparian factors is recommended to elucidate the specific spatial drivers of terrestrial diversity, particularly in temperate environments.

When riparian biodiversity was examined at broader spatial scales, we found strong variation across sites. Contrary to our predictions, the dissimilarity between assemblages at the same streams (β_3_) or distances (β_4_) was similar to the background site-level dissimilarity between all assemblages (β_1_). This indicates little spatial structuring among streams, with local environmental factors and the strong influence of distance from water determining community assembly [50]. When assemblages were pooled by the stream (β_2_), dissimilarity among assemblages was reduced, possibly as more regional biodiversity was captured when comparing larger assemblages. However, we found standing densities were lower in Brazil than in the UK, and β-diversity was higher and more turnover driven. This agrees with predicted higher predation and shorter development times in the warm tropics, increasing biomass turnover and reducing standing densities of invertebrates [19]. Lower densities increase the chance for rank-abundance changes and stochastic extinctions, while more generations per year increase random demography, all of which increase β-diversity in the tropics [19]. We did not observe higher α-diversity in the Brazilian assemblages, contrasting with our predictions based on common latitude-diversity patterns [29]. Nevertheless, the high β-diversity of the Brazilian sites, combined with low standing densities, implies that regional diversity was still higher in the tropical riparian ecosystems we studied.

The major differences in how riparian biodiversity is structured over space in the two regions have contrasting management implications. Multiple-site, landscape-level management is recommended to maintain the high turnover-driven β-diversity observed in Brazilian riparian ecosystems [26]. The high turnover with 150 m from water and apparent stochasticity in tropical riparian communities suggest a need for both wider buffers and protection of larger geographic areas to maintain the larger species pool and compensate for stochastic local extinction in small populations with low α-diversity. Our results support previous findings that emerging aquatic insects have a strong influence in the tropics [16], eliciting a strong response of terrestrial predators and potentially exerting an indirect top–down influence on other terrestrial preys [14], although other environmental gradients of riparian influence may also influence these patterns. Nevertheless, stream-based conservation approaches that boost populations of aquatic insects are predicted to have cascading impacts on riparian biodiversity in the tropics [51]. The greater reliance of predators on aquatic prey in tropical Brazil [16] also makes the wider terrestrial ecosystem more vulnerable from disruptions to aquatic–terrestrial linkages. This is expected to apply more generally to the tropics due to the lower tolerance of tropical ectotherms to rising environmental temperatures [52] and resource pressure from rapidly developing human populations [24]. By contrast, the abundance-driven β-diversity of temperate riparian ecosystems would benefit more from targeting the richest, most abundant communities at the expense of more depauperate communities [26]. In our study, these were the assemblages nearest water. However, the weak response of British spiders to proximity to water suggests this was driven by other gradients of aquatic influence, such as vegetation structure, water availability or microclimate [3], which should be explored in future studies. Thus, British riparian biodiversity is more likely to benefit from modification of the riparian environment directly than stream-based conservation measures that influence emerging insects. This may imply temperate ecosystems are less vulnerable to cross-ecosystem resource disruptions, potentially more vulnerable to alterations of terrestrial habitats [53], and that the UK’s current recommended buffer requirements should be increased to maximize terrestrial biodiversity provision [10].

Our results demonstrate that despite high heterogeneity and lack of clear spatial structuring across individual sites, riparian biodiversity is strongly impacted by the aquatic environment at local scales beyond stream borders and at broader scales between regions. Although some of the findings here may be applicable outside of Brazil and the UK, the environmental and biogeographical variation across tropical and temperate regions currently limits extrapolation to other areas. For example, research from a less seasonal temperate site found aquatic–terrestrial linkage strengths similar to that observed in tropical sites [16,54], suggesting climate seasonality may be more significant than the biogeographic realm [52]. While we took measures to control for the variation in seasonality between the two regions, our sampling was limited to a single season in both regions. Future research across multiple seasons is needed to fully elucidate how seasonal changes in the strength of aquatic–terrestrial linkages influence terrestrial biodiversity throughout the year. The comparative methodology employed here could be straightforwardly applied to other regions at different seasons to build a global understanding of riparian diversity. Nevertheless, the clear differences in riparian diversity patterns and distinct management recommendations between the tropical Brazilian and temperate UK sites found in this study highlight the issues with applying temperate findings to inform tropical conservation [8], the importance of examining diversity from multiple spatial scales [6] and the value of direct comparative methods in linking research from global regions.

## Data Availability

Data available from the Dryad Digital Repository [55]. Supplementary material is available online [56].
